# Decreased Number and Expression of nNOS-Positive Interneurons in Basolateral Amygdala in Two Mouse Models of Autism

**DOI:** 10.3389/fncel.2018.00251

**Published:** 2018-08-13

**Authors:** Xiaona Wang, Jisheng Guo, Yinsen Song, Qi Wang, Shunan Hu, Lingshan Gou, Yinbo Gao

**Affiliations:** ^1^Henan Provincial Key Laboratory of Children’s Genetics and Metabolic Diseases, Children’s Hospital Affiliated of Zhengzhou University, Zhengzhou, China; ^2^Center for Translational Medicine, The Sixth People’s Hospital of Zhengzhou, Zhengzhou, China; ^3^Department of Histology and Embryology, Guizhou Medical University, Guiyang, China; ^4^Center for Genetic Medicine, Xuzhou Maternity and Child Health Care Hospital, Xuzhou, China

**Keywords:** autism spectrum disorders, interneuron, nNOS, basolateral amygdale, mouse models

## Abstract

The basolateral amygdala (BLA) controls socio-emotional behaviors and is involved in the etiology of autism. We have recently shown that virtually every neuronal nitric oxide synthase (nNOS) positive cell is a GABAergic inhibitory interneuron in the mouse BLA. Here, stereology was used to quantify the number of nNOS-expressing interneurons in valproic acid (VPA)-exposed C57BL/6J (B6) and BTBR T^+^Itpr3^tf^/J (BTBR) mice models of autism. Additionally, the protein and mRNA levels of nNOS in the BLA were quantitatively assessed by western blot and qRT-PCR analysis, respectively. Our results showed the decreased number of nNOS interneurons in the BLA of animal models relative to autism. Consistently, nNOS was significantly reduced in the VPA-exposed and BTBR mice at both protein and mRNA levels. Together, these preliminary findings suggest that down-regulation of nNOS may be an attractive target for the pharmacological intervention in autism.

## Introduction

Autism spectrum disorders are a group of common neurodevelopmental disorders, characterized by repetitive behaviors, restricted interests, social deficits and communication difficulties (Lin et al., 2013). Altered function of several brain areas is thought to underlie the social and cognitive phenotype in autism. Identified brain regions include the amygdala, prefrontal and temporal cerebral cortex and hippocampus, striatum, among others (Sosa-Díaz et al., 2014; Hashemi et al., 2017). Accumulating evidence suggests loss of GABAergic cells in the hippocampus and cerebral cortex of autism (Sgadò et al., 2013; Sabanov et al., 2017; Ariza et al., 2018). Noteworthy, it is reported that morphology and neurophysiology change in the amygdala of autism (Markram et al., 2007; Bringas et al., 2013). Recent efforts have shown the remarkable decrease in neuronal numbers in the lateral nucleus amygdala of autistic subjects (Varghese et al., 2017). Studying in animals has confirmed that GABAergic interneurons within the basolateral amygdala (BLA) are strongly implicated in autistic-relevant behaviors (Prager et al., 2016). Collectively, these results demonstrate that BLA dysfunction may contribute to social and emotional disturbance in autism (Todd and Anderson, 2009; Lin et al., 2013).

Emerging evidence suggests that deficits in GABAergic inhibitory interneurons are the pathophysiological mechanism of autism (Dong et al., 2016; Lee and Kim, 2017). In particular, glutamic acid decarboxylase 65 and 67 (GAD_65_, GAD_67_) are reduced in the cortex of valproic acid (VPA)-treated animals (Oblak et al., 2011a). Studies have also shown decreased GAD_65_ and GAD_67_ mRNA levels of the cerebellum in autistic patients (Yip et al., 2007, 2009). Remarkably, it has been established that the subpopulations of GABAergic interneurons in the mouse BLA consist of calcium-binding proteins (parvalbumin (PV), calbindin and calretinin, neuropeptide Y and neuronal nitric oxide synthase (nNOS); Wang et al., 2017). At the behavioral level, mice with reduced PV interneurons expression display the robust autism-relevant behaviors (Filice et al., 2016). The reduced number of PV-containing cells could disrupt the balance of excitation/inhibition and alter gamma wave oscillations in the cerebral cortex of autistic subjects (Hashemi et al., 2017). Likewise, evidence has accumulated the lower density and number of calretinin, calbindin and neuropeptide Y interneurons in autism (Oblak et al., 2011b; Peñagarikano et al., 2011; Adorjan et al., 2017). Notably, we recently found that virtually every nNOS-positive cell is GABAergic interneuron in the BLA (Wang et al., 2017). Despite these observations, it remains presently unknown whether nNOS-expressing interneurons are affected in the BLA of autism.

Environmental exposure and genetic susceptibility are increasingly being recognized as potential risk factors for autism. More specifically, VPA as antiepileptic medication during pregnancy, exhibits similar to the core symptoms of autism, including impaired social interaction, stereotypic/repetitive behaviors and sensory/communication deficits (Roullet et al., 2013; Nicolini and Fahnestock, 2018). Meanwhile, BTBR T^+^Itpr3^tf^/J (BTBR), a well-studied mouse model of idiopathic autism, displays repetitive self-grooming, deficiencies in social interactions, as well as minimal vocalization in social settings (McFarlane et al., 2008; Provenzano et al., 2016).

Therefore, in the present study, we were prompted to investigate nNOS-expressing interneurons counts in the BLA with stereological techniques in VPA-exposed and BTBR mice. Additionally, nNOS protein and mRNA levels were quantitatively determined by western blot and qRT-PCR, respectively.

## Materials and Methods

### Animals

All procedures were approved by the Shandong University Animal Care and Use Committee and were carried out in compliance with the National Institutes of Health guide for the care and use of Laboratory animals (Publication No. 85-23, revised 1985). BTBR mice were obtained from Model Animal Research Center of Nanjing University. C57BL/6J (B6) mice were provided by Shandong University. Mice, 8 weeks old, were housed in plastic cages (30 cm wide × 18 cm long × 14 cm high) in humidity (30%) and temperature (23°C) maintained under a 12:12 h light/dark cycle (lights on at 07:00). Food and water were available *ad libitum*. All efforts were made to minimize the animal suffering and the number of animals used.

### Drug Administration and Experimental Groups

According to the studies of Wu et al. (2017), single female and male B6 mice were randomly allocated to mate overnight. Pregnancy was determined by the presence of a vaginal plug on embryonic day 1 (E1). We removed male mice from cages in the same day. Pregnant mice received an intraperitoneal injection of 600 mg/kg VPA sodium salt (Sigma Aldrich, St. Louis, MO, USA) on E13 (Al-Askar et al., 2017). Control mice received injection of equal volumes of saline. Pup mice were weaned on postnatal day 21 (P21) and housed in groups of four per cage. Besides, B6 animals have been routinely used as the control for the BTBR mice in autism-related studies (Cheng et al., 2017; Meyza and Blanchard, 2017). Only male offspring were used in this study.

On postnatal 35 days, four offspring groups were deeply anesthetized with pentobarbital sodium, decapitated and processed for stereology, western blot and real time-PCR analysis.

### Immunohistochemistry Staining

Immunohistochemistry was conducted as we have previously described (Wang et al., 2017). Adult mice were intracardially perfused with 4% paraformaldehyde and the brain was removed immediately. The BLA was postfixed for overnight, successively placed in 30% sucrose in 0.1 M phosphate-buffered saline (PBS) overnight and cut on a sliding microtome (40 μm). Free floating sections were quenched for 30 min in 3% H_2_O_2_/30% methanol in PBS and incubated in a blocking solution (5% normal goat serum and 2.5% bovine serum albumin in PBST (0.25% Triton X-100)) for 30 min at room temperature. Sections were then incubated with the goat anti-rabbit nNOS (1:5000; Sigma) in the blocking serum at 4°C overnight. After rinsing in PBST (three times for 10 min), sections were reacted for 2 h at room temperature with biotinylated secondary antibody goat anti-rabbit IgG (1:500, Vector Laboratories; Burlingame, CA, USA) and incubated with avidin biotin peroxidase complex (Vector ABC Kit, Vector Laboratories). The reaction was visualized by detection solution (0.25 mg/ml 3,3′-diaminobenzidine in combination with 0.03% H_2_O_2_ in PBS). Sections were then transferred onto mounted on glass slides, dehydrated with ascending alcohols, rinsed in xylene and cover slipped.

### Stereological Quantification

To determine the total number of nNOS-positive interneurons in the BLA in two mouse models of autism, we used the optical dissector principle as previously described (Wang et al., 2017). Briefly, sections were outlined with Stereo Investigator software (MBF Biosciences, Williston, VT, USA). Brain regions of interest (ROIs) were determined based upon stereotactic coordinates provided by the Franklin and Paxinos atlas (Franklin and Paxinos, 2007) at 0.94–2.18 from bregma for the BLA. A 1-in-6 series of sections was analyzed and the counting frame was 40 × 40 μm. The dissector height was 13 μm. Section thickness was measured for every section counted per mouse (17 μm). A nNOS-containing cell body was counted as it came into focus within the dissector box and above the bottom exclusionary plane. Criteria for counting cells required that interneurons displayed nNOS-expressing immunoreactivity and morphological features in line with each cell type. The total number N of nNOS-expressing cells in the ROIs was calculated using the formula:

N=ΣQ*t/h*1/asf*1/ssf

where Σ*Q* is the total number in the BLA acquired with the optical dissect, *t* is the mean thickness of section, h is the optical dissector height, as f represents the area sampling fraction, and ssf is the fraction of section sampling. Images of sections were captured with a Nikon TE2000U microscope.

### Western Blot Analysis

Western blot analysis was carried out as we previously described (Wang et al., 2014). In brief, BLA of homogenates were centrifuged for 15 min at 10,000 *g* at 4°C remove any cell debris. Proteins (30 μg per well) were separated by sodium dodecyl sulfate-polyacrylamide gel electrophoresis (100–120 V) and transferred to polyvinylidene difluoride membranes (Millipore, Milford, MA, USA). The membranes were blocked with 5% non-fat milk powder in Tris buffered saline containing 0.1% Tween 20 (TBST) for 60 min at room temperature. Subsequently, the membranes were probed with the primary antibodies overnight at 4°C: nNOS (1:5000; Sigma) and GAPDH (1:1000; CST, Beijing, China). The membranes were incubated with the secondary antibody horseradish peroxidase conjugated to IgG (1:1000; ZSGB-Bio, Beijing, China) and washed three times with TBST for 10 min each. Protein bands were visualized using the super enhanced chemiluminescence reagent (Pierce, Rockford, IL, USA) and images were captured by luminescent image analysis system (Fujiflm, LAS-4000 mini, Japan). Bands intensities were determined by Image J software program.

### Quantitative Real-Time PCR

Total RNA (500 ng) extracted from BLA was isolated using TRIzol reagent (Invitrogen, Carlsbad, CA, USA). RNA concentrations were determined by a NanoDrop spectrophotometer (Thermo Scientific, Wilmington, MA, USA). First-strand cDNA synthesis was performed using Promega’s reverse transcriptase kit (Promega, Southampton, UK) following manufacturer’s instructions. qRT-PCR was performed to examine the expression of mRNA of the nNOS and GAPDH genes using the universal KAPA SYBR FAST qPCR Universal Master Mix (Kapa Biosystems, Woburn, MA, USA). The sequences of the primers used were as follows: nNOS forward 5′-ACC CAA CGT CAT TTC TGT CC-3′ and reverse 5′- AAG GTG GTC TCC AGG TGT GT-3′; GAPDH forward 5′-CGGAGTCAACGGATTTGGTCGTAT-3′ and reverse 5′-AGCCTTCTCCATGGTGGTGAAGAC-3′. Gene expression quantitation was carried out in a DNA thermal cycler (Bio-Rad, San Diego, CA, USA) as follows: a denaturation step of 95°C for 15 min, 50 cycles of denaturation at 94°C for 15 s and annealing/extension/data acquisition ranging from 60°C to 72°C for 20 s. The housekeeping gene GAPDH mRNA was used as an internal reference to normalize the mRNA content for each sample. Gene expression data were expressed as a proportion of GAPDH gene, as a reference, using a 1/ΔCt calculation.

### Statistical Analysis

Statistical analysis was performed with GraphPad Prism 5.01 (GraphPad Software, San Diego, CA, USA), using the Student’s *t* test. The level of statistical significance was set at* p* < 0.05.

## Results

### Stereological Quantification

**Figure 1:**
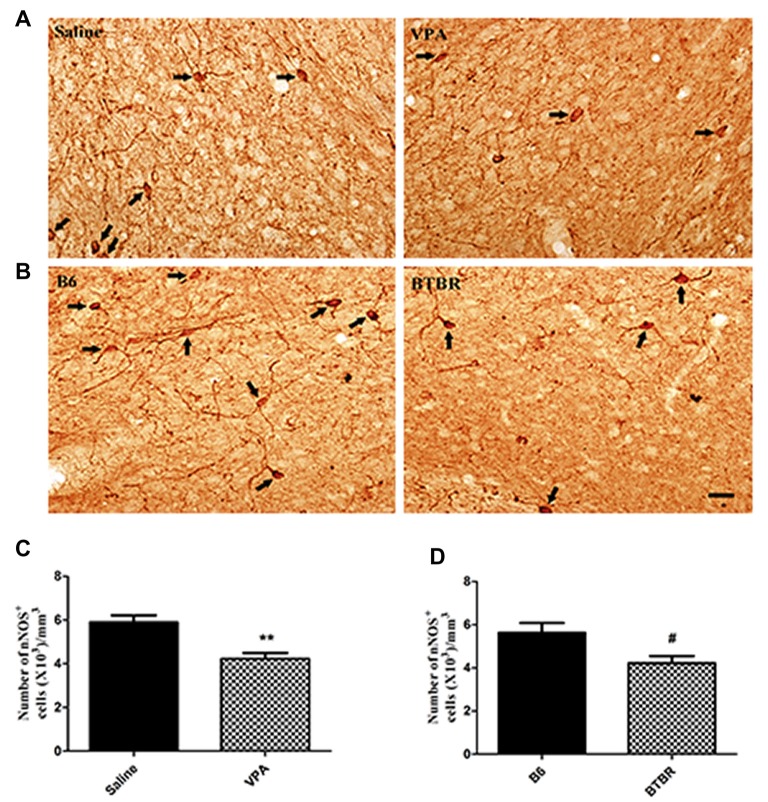
Stereological estimations of neuronal nitric oxide synthase (nNOS)-expressing interneurons in valproic acid (VPA)-exposed **(A)** and BTBR T^+^Itpr3^tf^/J (BTBR; **B**) mice in comparison to those in saline-treated and C57BL/6J (B6) mice, respectively. Some nNOS immunoreactive interneurons have been pointed with arrow. Significant differences are observed in nNOS-containing cells in two animal models of autism **(C,D)**. **p < 0.01 vs. saline-treated mice. ^#^*p* < 0.05 vs. B6 mice. Scale bars: 25 μm.

To determine the extent of nNOS-expressing cell loss in the current study, we first counted the total number of nNOS-containing interneurons in the VPA and BTBR mice models of autism. As illustrated in Figure 1, the total number of nNOS interneurons in the BLA of saline-treated offspring, estimated by stereologically counting nNOS-positive interneurons, was (5.63 ± 0.92) × 10^3^/mm^3^ (*N* = 8). However, in the VPA-induced autism model, the total number of nNOS-containing cells was (4.23 ± 0.75) × 10^3^/mm^3^, a 15% reduction compared to B6 controls (*N* = 8, *p* < 0.01, Figures 1A,C).

Similarly, the total number of nNOS-immunoreactive interneurons in the BLA of B6 group was (5.63 ± 1.30) × 10^3^/mm^3^ (*N* = 8). In the BTBR mice, the total number of nNOS-expressing cells was (4.23 ± 0.92) × 10^3^/mm^3^, a 43% reduction as compared to B6 strains (*N* = 8, *p* < 0.05, Figures 1B,D), indicating that nNOS-containing interneurons in the BLA are vulnerable to VPA-exposed and BTBR mice.

### Alterations in Protein and Gene Levels of nNOS

**Figure 2:**
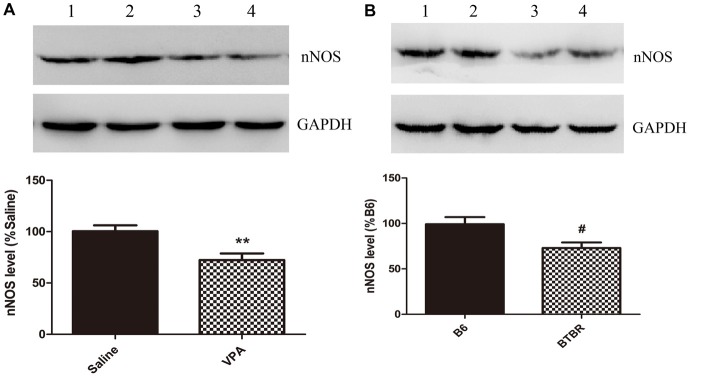
Quantitative western blot analysis of nNOS expression in basolateral amygdala (BLA) samples of VPA-treated and BTBR mice. A representative western blot and the quantification of nNOS protein levels in VPA-exposed **(A)** and BTBR **(B)** mice are shown. **(A)** Band 1, 2 saline-treated group; Band 3, 4 VPA-exposed group. **(B)** Band 1, 2 B6 group; Band 3, 4 BTBR group. Data are from six independent experiments and are shown as mean ± SEM. Results are expressed as a percentage of normalized nNOS levels measured in controls, defined as 100%. GAPDH signals served as loading controls and were used for the normalization of the nNOS signals. **p < 0.01 vs. saline-treated group. ^#^*p* < 0.05 vs. B6 group.

These nNOS-positive interneurons are suggested to play a crucial role in the regulation of neuronal excitability in the BLA (Mańko et al., 2012). Next, we compared the nNOS protein levels in the VPA mouse model of autism with those in saline-exposed offspring. As shown in Figure 2, the results showed that exposure of mice to VPA significantly decreased nNOS protein expression in the BLA compared with the corresponding controls (*N* = 6, *p* < 0.01, Figure 2A). Likewise, nNOS expression of VPA-treated mice was remarkably decreased as compared to B6 mice (*N* = 6, *p* < 0.05, Figure 2B). Thus, nNOS protein expression was down-regulated in nNOS-positive GABAergic cells in the BLA of VPA-exposed and BTBR mice.

**Figure 3:**
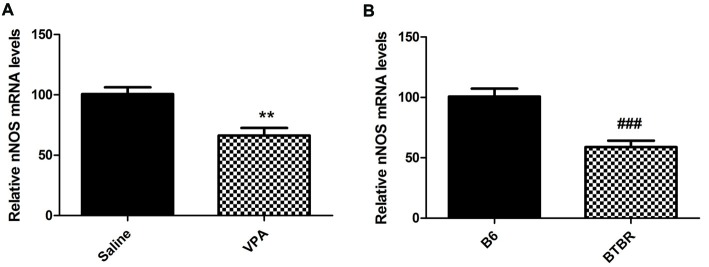
qRT-PCR values from VPA-treated **(A)** and BTBR **(B)** mice representing nNOS mRNA levels were normalized to GAPDH mRNA levels and expressed as fold change compared to controls. Data from six independent experiments were pooled together and are shown as mean ± SEM. **p < 0.01 vs. saline-treated controls. ^###^*p* < 0.001 vs. B6 mice.

Furthermore, our results demonstrated that mice treated with VPA markedly exhibited lower nNOS mRNA levels when compared to saline group (*N* = 6, *p* < 0.01, Figure 3A). As expected, nNOS mRNA expression in the BLA of VPA-treated offspring was dramatically decreased compared with B6 mice (*N* = 6, *p* < 0.001, Figure 3B). Thus, the nNOS mRNA was dramatically reduced in the nNOS-expressing interneurons in the BLA of VPA-treated and BTBR mice.

## Discussion

Our study demonstrated that number of nNOS interneurons in BLA was reduced in two well-established mouse models of autism. Furthermore, nNOS expression was remarkably decreased in VPA-exposed and BTBR mice at both protein and mRNA levels.

Converging evidence indicates altered GABAergic signaling is related to autism, culminating in the proposal that increase in excitation/inhibition ratio is critical cause of autism (Han et al., 2012, 2014; Lee and Kim, 2017). Specifically, postmortem neuropathological studies of individuals with autism have shown decreased GABA precursors in certain neuron populations and reduced number, density and expression of GABA receptors in the cortex (Blatt and Fatemi, 2011). Moreover, structural amygdala abnormalities such as reduced neuron number and changes in volume have been observed in individuals with autism (Schumann et al., 2004; Schumann and Amaral, 2006). It has been widely reported that decreasing GABA function decreases sociability in the BLA (Paine et al., 2017). Numerous studies have demonstrated that prenatal exposure to VPA and BTBR mice lead to autism phenotype, which can be used to model the complexity of autism symptoms (Han et al., 2014; Barrett et al., 2017). Accordingly, we first ascertained by stereology that the number of nNOS-expressing cells was altered in the BLA of VPA-exposed and BTBR mice. Of particular noteworthy, our data showed that there was indication of the decrease of nNOS-expressing cells in the BLA of autism, consistent with previous results obtained in the decreased number and density of PV interneurons in the prefrontal cortex in autism (Filice et al., 2016; Ariza et al., 2018). The reduced number of PV-immunoreactive cells could disrupt the balance of excitation/inhibition and elicit core deficits of autism (Wohr et al., 2015; Hashemi et al., 2017). Of relevance, animal models that exhibit an imbalance in the ratio of pyramidal cells to interneurons in the cortex show core autism-related deficits, including abnormalities in reciprocal social interactions and stereotyped behaviors (Helmeke et al., 2008). It is therefore possible that disruptions in the BLA region through synaptic connectivity formed by reduction of nNOS interneurons may result in disrupted socio-emotional behaviors of autism. Additional researches including direct recordings from nNOS interneurons during the behavioral experiment in autism and observing autism-related phenotypes after modulating the excitation/inhibition balance due to decreased nNOS levels could give us the opportunity to prove the relationship between the two.

While an involvement of nNOS interneurons in autism is rather undoubted, it remains unclear whether the observed reduced nNOS-expressing cells in the BLA of autism are the result of: (I) a truly decreased number of nNOS-positive interneurons due to developmentally immature or perturbed state (e.g., region-improper localization of nNOS-containing cells, enhanced susceptibility, precursor and premature neuronal death) or (II) alternatively from the reduction of nNOS protein (or mRNA) levels or the inability to express sufficient levels of the protein (or mRNA). To resolve this question, one needs to clarify whether the decrease in nNOS-expressing cells in two canonical autism mouse models might in part the result of nNOS down-regulation. Noticeably, the present data showed the decrease in nNOS protein expression for nNOS-positive cells in the VPA-exposed and BTBR mouse models. The similar magnitude in down-regulation of nNOS mRNA manifests the regulation at the level of transcription, in agreement with the reduced GABA content, GAD_65_ and GAD_67_ mRNA levels in autism (Chao et al., 2010). Future investigation is necessary to identify possible mechanisms for nNOS interneurons vulnerability and to illustrate the functional consequences for potentially developing autism-like features.

Accumulating evidence indicates that nNOS-containing cells are implicated in inhibitory synaptic transmission (Armstrong et al., 2012; Lange et al., 2012). Particularly, Li et al. (2014) reported that firing of hippocampal nNOS interneurons creates suppression of synaptic inhibition. Previous work has revealed that nNOS-expressing cells evoke a slow inhibitory postsynaptic current (IPSC) caused by volume transmission of GABA and modulate neurons slow inhibition in the BLA (Capogna and Pearce, 2011; Mańko et al., 2012). It should be noted that nNOS-mediated long-term regulation of inhibitory transmission might contribute to fear learning in amygdala (Lange et al., 2012). Following this line of reasoning, thus, identification the slow inhibition properties of nNOS-containing cells within the BLA is crucial for understanding how emotional and cognitive impairments in addition to autistic symptoms are processed. Consequently, the demonstration that nNOS levels are reduced may suggest a shift in the excitation/inhibition balance to a reduced inhibition, considering the proven function of nNOS in synaptic transmission on autism-related defects.

In a complementary fashion, one limitation is that molecular alterations in the VPA model are similar but not identical to those demonstrated in individuals with idiopathic autism (Nicolini and Fahnestock, 2018). On the other hand, the striking feature of BTBR mice is lack of corpus callosum, which is rare among autistic individuals (Meyza et al., 2013). Disc1 mutation, carried by BTBR mice, has been related with autism in some cases. Indeed, a substantial body of evidence indicates that a child is predisposed to autism due to dynamic interplay between environment and genetics (Olexová et al., 2012). Therefore, to further explore the function of nNOS-expressing interneurons in autism, more attention is needed for the development of a combined-model approach, in which the core symptoms and etiological character of autism are represented.

To conclude, the present study has indicated the decrease in the number and expression of nNOS-positive interneurons in autism, which is a fundamental step that will shed light on the origins of altered excitation/inhibition balance in autistic amygdala. As well, testing whether approaches aimed at restoring normal nNOS expression levels and/or nNOS interneuron function might ameliorate autism relevant behavioral phenotypes appears therefore warranted and may represent potential promise for novel therapeutic strategies.

## Author Contributions

XW and YG designed the research and wrote the manuscript. JG, YS, QW, SH and LG conducted experiments and analyzed the data.

## Conflict of Interest Statement

The authors declare that the research was conducted in the absence of any commercial or financial relationships that could be construed as a potential conflict of interest.
